# 3D-based buccal augmentation for ideal prosthetic implant alignment—an optimized method and report on 7 cases with pronounced buccal concavities

**DOI:** 10.1007/s00784-022-04369-1

**Published:** 2022-01-22

**Authors:** Hans-Joachim Nickenig, Maximilian Riekert, Matthias Zirk, Max-Philipp Lentzen, Joachim E. Zöller, Matthias Kreppel

**Affiliations:** Department of Oral and Maxillofacial Plastic Surgery and Interdisciplinary Department of Oral Surgery and Implantology, University of Cologne, Kerpenerstrasse 32, 50931 Cologne, Germany

**Keywords:** Titanium mesh, Screw-retained restoration, CBCT, CAD-CAM technology

## Abstract

**Objectives:**

Screw-retained restoration of implants is advantageous for biological and esthetic reasons. Due to buccal concavities, however, this preferred type of restoration can only be used in about half of the anterior indications. Based on case series, an optimized method for the treatment of such indications is to be described; the clinical reliability is to be ascertained by means of measurements (before and after augmentation) and assigned to the current literature.

**Material and methods:**

A case series of seven cases with buccal concavities of the anterior alveolar ridge were treated with optimized method, which is presented step-by-step until the prosthetic restoration. The depths of the bone concavities were measured and related to the bone gain after augmentation procedure respectively after implantation.

**Results:**

Linear measurements of the buccal concavities showed an average undercut of 4 mm [SD ± 1.13]. After healing period of six months, the buccal concavities could be compensated bony to such an extent that implants could be inserted in correct position and angulation. On average, there was a horizontal bone gain of 3.7 mm [*SD* ± 0.59]. Even after implantation and another six months of healing, stable bone dimensions could be assumed with an average of 4.3 [*SD* ± 0.83] mm of bone gain compared to baseline. In six of the seven cases, the favorite screw-retained, one-piece full-ceramic restoration could be fixed on the implants. Due to the implant axis, one case had to be treated with a cemented two-part full-ceramic system.

**Conclusions:**

With the described optimized method the most favorable screw-retained restoration can also be used in situations with unfavorable concavities of buccal bone. Especially for this indication, a special form of the horizontal deficit, the customized bone regeneration with titanium meshes is highly reliable in terms of healing and extent of augmentation. However, long-term results and a study/control group are required to evaluate the effectiveness of the presented protocol.

Clinical relevance.

Since these situations require an augmentation that is up to 5 mm thick and a procedure that is as minimally invasive as possible appears to be necessary in the visible area, an optimized method is described in this publication.

## Introduction

Long-term data on the esthetic outcome of anterior implants placed in fully healed bone without hard or soft tissue grafting have shown that the lack of sufficient buccal hard and soft tissue in the horizontal dimension is cited as one of the most common problems [1, 2]. There are many clinical methods of repairing alveolar bone defects, including guided bone regeneration (GBR), onlay bone grafting, and bone splitting and spreading. Due to the minimally invasive technique, the multidirectional osteogenesis capability and osteogenic stability, GBR is one of the most frequently used methods to compensate for horizontal defects [3, 4]. The theory of GBR technology is to selectively prevent epithelial cell and connective tissue migration from the bone defect area through the barrier membrane, allowing osteoblasts to preferentially enter the defect situation to complete bone induction and regeneration [5–7]. In the meantime, studies have shown that in the clinical application of GBR, spatial support of the bone defect can play a more important role than cell-selective isolation [8].

In severe defects of the alveolar bone, GBR with titanium meshes showed superior mechanical and osteogenic properties compared to conventional GBR with absorbable and non-absorbable membranes [9, 10]. Commercially available titanium meshes originally used, had to be bent and shaped manually during the operation in order to adapt them to the alveolar ridge. This process is often associated with various disadvantages, such as an imprecise fit, the risk of infection and exposure of meshes due to sharp edges [11]. Due to the advances in digitalized dental technologies, custom-made titanium meshes are primarily used today. Systematic reviews showed that personalized (custom-made) titanium mesh allow to shorten the operation time, reduce the occurrence rate of bone augmentation complications, and improve the success rate of surgery [11–13]. Using GBR with 3D-based titanium mesh, most researchers achieved an average bone augmentation of 4–5 mm [14–16]. In the case of a one-stage procedure with implantation in the same session, a slightly lower bone gain can be assumed with an average of 3–4 mm [17, 18].

With regard to the aetiology of different defects, it should be noted that in terms of dimensional changes of the alveolar ridge, bone resorption—especially of the buccal bone plate—is regularly observed after extraction of teeth [19, 20]. Cologne classification of the alveolar ridge defect (CCARD) divides the extent of these horizontal bony defects up to 4 mm, 4–8 mm, and more than 8 mm [21].

In a study on human specimens, it was found for the anterior region of the upper jaw that up to 4 mm deep buccal concavities are to be expected in over 50% of cases. The highest rate of buccal undercuts was detected in the area of ​the lateral incisors [22]. In a simulation study with virtual implant placement, Chan et al. [23] investigated the risk of implant buccal plate perforation in the esthetic zone and found high incidence (approximately 20 to 30%) of fenestration if an implant is placed in prosthetically correct position and direction. Consequently, the axis of the implant and that of the restoration do not coincide, which makes it often impossible to use a screw-retained restoration. Screw-retained reconstructions exhibited less biological problems like implant failures or marginal bone loss than cemented reconstructions [24].

Peri-implant diseases are prevalent with a weighted mean prevalence rate of 43% across Europe and 22% across South and North America. Although the main etiologic agent is bacterial biofilm, a myriad of factors influence the initiation and progression of the disease [25]. To reduce the risk of peri-implant disease, associated with excess cement, a crown margin at the level of the marginal mucosa providing sufficient access is recommended only for the posterior region. Since it is often not possible to place the crown margin at mucosal level in the visible area, a screw-retained reconstruction is preferred for the esthetic zone whenever possible [26]. Therefore cementation should be avoided where possible [27].

Damage to the critical anatomic structures during dental implant placement can lead to serious complications. These complications usually occur when the anatomic features such as alveolar ridge contour are not properly investigated prior to the surgery. Therefore, having proper knowledge, e.g. with three-dimensional diagnostics of the specific anatomic factors, is key to avoiding intra- and postsurgical complications [28].

Angled screw channel abutments allow access to the screw at an angle of up to 25 degrees relative to the implant axis. These systems have proven to be a practicable therapy option for implants that cannot be placed in the ideal three-dimensional position in the anterior region [29]. Limiting the system is the additional space requirement, which increases the risk of soft tissue complications, and the need for a more voluminous screw channel that enables the screwdriver to be inserted correctly, which can lead to a thinning of the veneering materials and possibly to technical or esthetic problems [30]. Similar problems arise for horizontal screw connections and cross-pin retained restorations [31].

Surgical-prosthetic protocols for the therapy of the bony concavities of buccal bone in esthetically relevant region, in order to enable a suitable three-dimensional prosthetic alignment, have either not been developed or are not yet known. For these reasons, the aim of this study is to describe an optimized method with 3D-based titanium meshes for situations with bony concavities on the buccal side of the anterior alveolar ridge, with which it is possible to design ideal implant restorations without prosthetic compromises or restrictions.

## Material and methods

### Linear measurements of buccal concavities and bone gain

Most of the morphological studies investigated the lingual undercut and incidence of lingual plate perforation during implant bed preparation in the mandible [32, 33]. In a simulation study with virtual implant placement, Chan et al. [23] investigated the risk of implant buccal plate perforation in the esthetic zone and found high incidence (approximately 20 to 30%) of fenestration if an implant is placed in prosthetically correct position and direction. The highest rate of buccal undercuts was found in the region of lateral incisors. In a cadaver study, the deepest concavities for the lateral incisor sites were measured with 3.15 mm (+ / − 1.32) [22]. For this measurement, the most anterior point (P) of the alveolar bone on the buccal surface was connected with a reference line (R) to the nasal floor parallel along the alveolar ridge, at the midline of the selected root (ideal virtual implant direction) (Fig. 1.1). If the remaining bone is used, due to the inclination of the implant towards the oral cavity, only an implant of smaller diameter and a two-part prosthetic restoration can be used (Fig. 1.2). If a screw-retained, one-piece restoration favored for the esthetic area is to be used in these situations, augmentation is required (Fig. 1.3).

For measurement of bone gain the method described above was modified. For this purpose, the reference line (R) is placed on the surface of the newly acquired buccal bone and the distance to the previously determined concavity depth now corresponds to the bone gain.

### Literature search

**Fig. 1:**
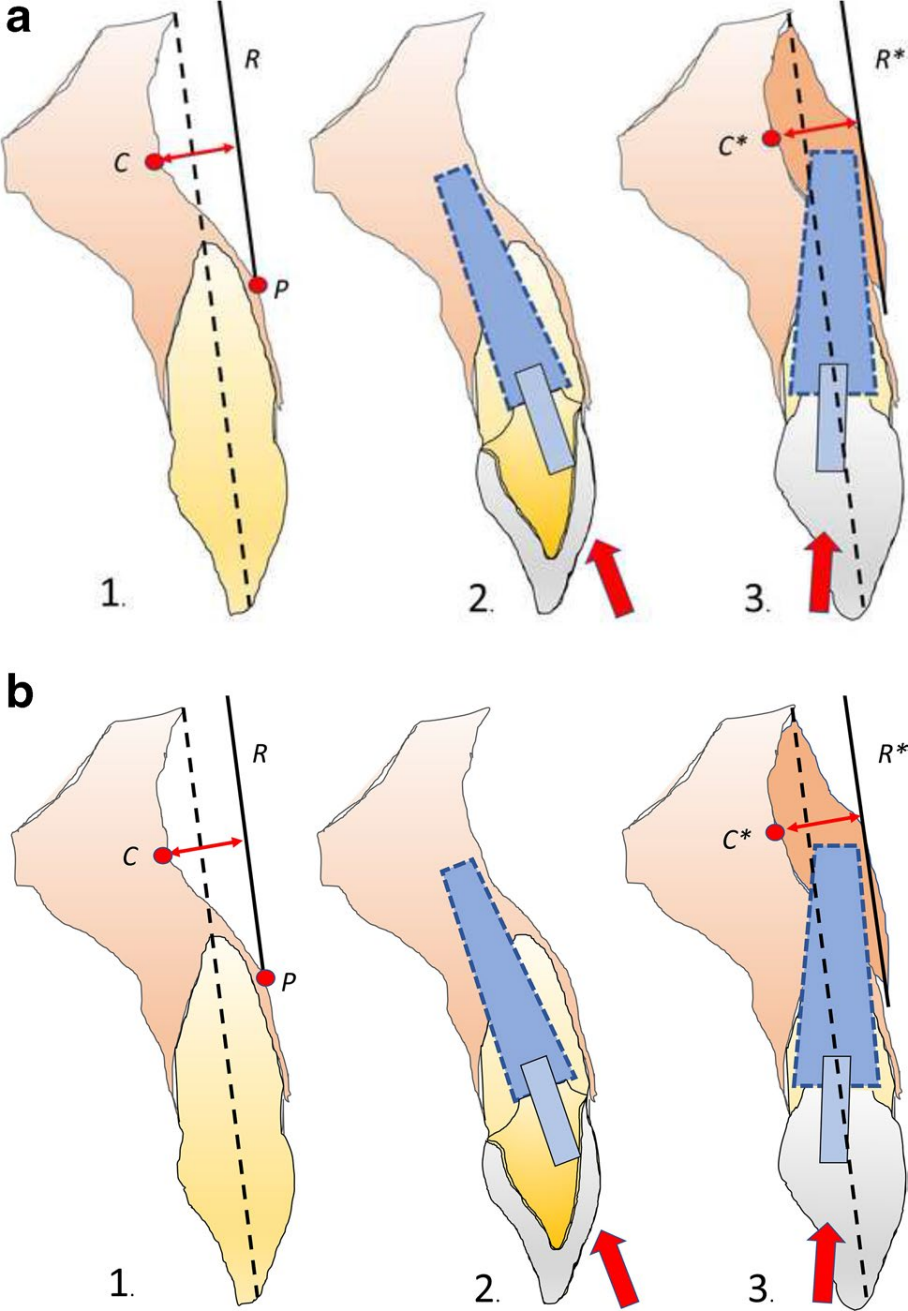
(1.1) Measurement of concavity depth (C) with the most anterior point (P) and the reference line parallel to midline of selected root (R); (1.2) inclining the implant while using the residual bone (1.3) augmentation of concavity which allows implant with wide diameter in regular direction and one-piece, screw-retained restoration. Right drawing with modification of the method for measuring bone gain

A survey of the literature, without limitation regarding the year of publication, was conducted using the medical databases MEDLINE/PubMed. Articles that were published before July 1, 2021, were included. The search strategy was limited to in vitro, in vivo, and human studies that reported data on GBR with titanium meshes. Studies using a barrier membrane for treatment of periodontal defects (guided tissue regeneration (GTR)) or peri-implantitis were excluded Fig. 1.

### Case series

**Fig. 2:**
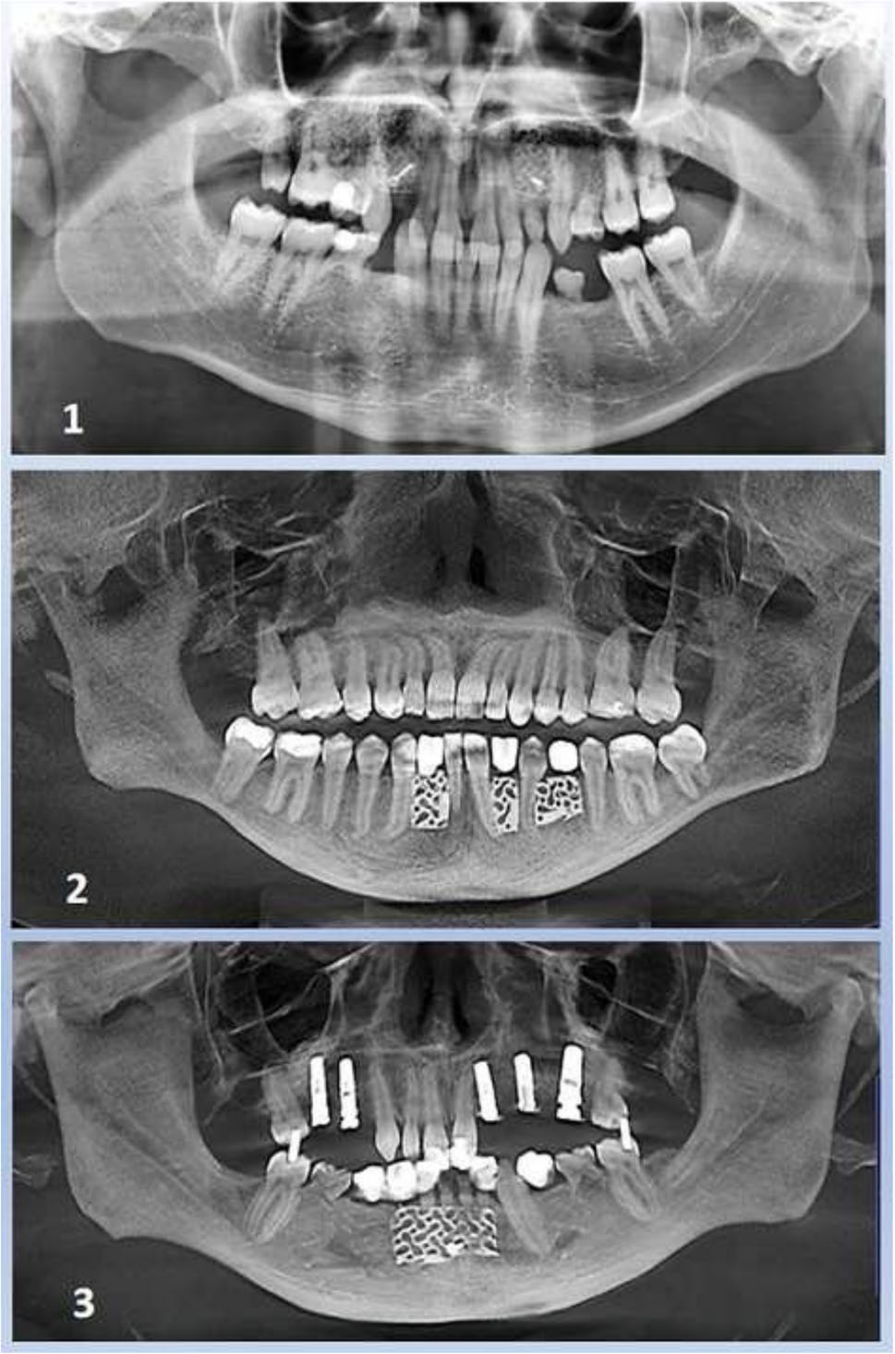
Panoramic views to the case series 1–3: patient 1 with missing teeth and need of augmentation in the canine region (tooth number 13, 23 (FDI)), patient 2 with three single tooth gaps in the lower jaw, patient 3 with a wide dental gap in the lower jaw and severe bone defects with concavities on the buccal side of the alveolar ridge

In the following, 7 surgical sites in 3 patients were enrolled in this case series (Fig. 2). The patients were referred to the Department of Oral and Maxillofacial Plastic Surgery of the University of Cologne with an indication for implants in situations with bony concavities on the buccal side of the anterior alveolar ridge in upper and lower jaw.

The first patient, a 28-year-old man was complaining about two missing teeth in the canine region (tooth number 13, 23 (FDI)), which caused esthetic deficits and difficulties in speech. He also reported chewing problems due to further inherited non-placements of teeth in the lower jaw (tooth number 34, 35, 44, 45). In the following, this case is discussed in detail to illustrate the method step-by-step.

Second patient, a 23-year-old woman, had three single tooth gaps (34, 32, 42) in the lower jaw due to inherited non-placements of teeth. After orthodontic treatment, her main problem was an esthetic one. The alternative therapy of restoration with fixed partial denture was not indicated due to the clinically intact neighboring teeth. The third patient, a 20-year-old woman, had severe bone defects with concavities on the buccal side of the alveolar ridge in the anterior mandible. For restoration with a fixed partial denture, implants were planned in region of tooth number 32 and 42.

The three patients did not have a history of smoking or any systemic disease.

### Radiographic imaging

For the anterior regions in the upper and lower jaw, the cross-sectional views of the CBCT (cone beam computed tomography, Galileos Comfort; Sirona Dental Systems, Bensheim, Germany) indicated the alveolar ridge as a typical undercut type with labial concavities of a depth of about 4 mm, thus requiring augmentation for an adequate implant placement with the prosthetic goal of screw-retained full-ceramic restorations.

Before augmentation, a baseline measurement of concavity depth was performed for each of the seven situations using the method described above.

The time for the first CBCT scan was before the augmentation to determine the concavity depth and to plan the 3D-based titanium mesh. Second timepoint of CBCT scan was after 6 months of healing period to plan the removal of titanium mesh and 3D-guided implant placement. The final CBCT scan was carried out in order to check quantity and quality of sensitive buccal bone after a further 6 months of healing period.

### Fabrication of the 3D-based titanium meshes

After acquisition of a CBCT dataset, 3D-projections of the atrophied regions were obtained by using a reverse engineering software (Yxoss CBR®, Filderstadt, Germany [34, 35]. The necessary bone volume was added digitally and the individualized titanium meshes were designed. The inner contour of the lattice structure represented the desired augmentation volume. By using computer-aided design/computer-aided manufacturing (CAD/CAM) procedures and rapid prototyping, the final design was achieved and confirmed interactively by the surgeon. After the 3D-printing process, the titanium meshes were sterilized and sent to our clinic.

### Protocol of augmentation procedure with CAD-CAM titanium meshes

**Fig. 3:**
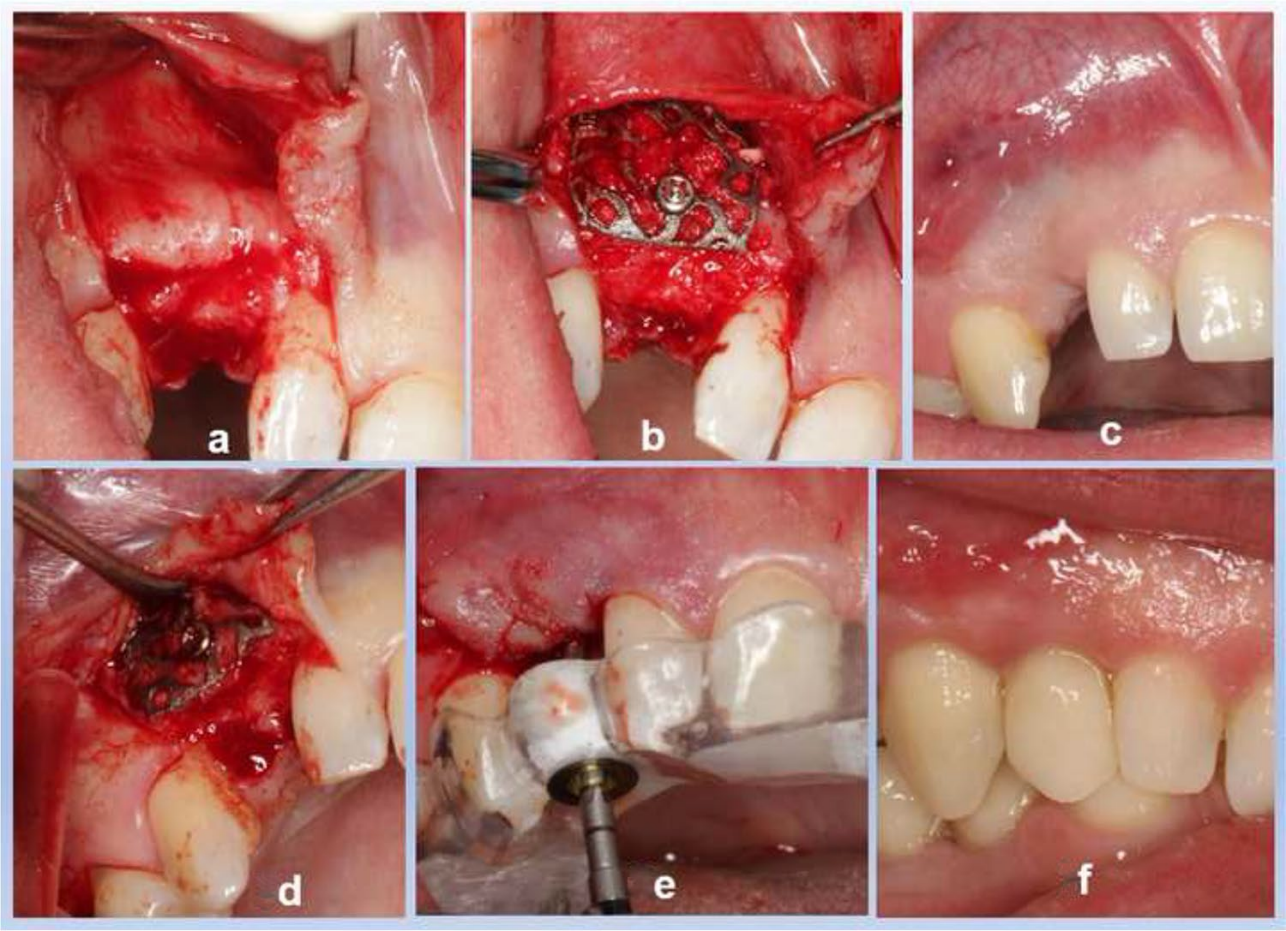
**a**–**f** Combined surgical-prosthetic procedure: **a** Mobilization of mucoperiosteal flap, view to the labial concavity, **b** fixed and bone-filled titanium mesh, **c** after 6-month healing period, **d** re-opening and removal of titanium mesh, **e** guided implant surgery, **f** after second healing period: prosthetic treatment with final one-piece, screw-retained restoration

The described method of 3D-based buccal augmentation for ideal prosthetic implant implements a two-stage protocol, where step 1 is augmentation and step 2 is guided implant placement. The augmentation procedure is first described below:

All surgeries were performed under local anesthesia. In order to damage the soft tissue as little as possible, incisions were made by means of a crestal incision and posterior relief. After the preparation, the mucoperiosteal flap was positioned in a manner comparable to a door opening (Fig. 3a). The meshes were filled by using a mixture of autologous bone graft and bone substitute biomaterial (Bio Oss®, Geistlich, Wolhusen, Switzerland) in a 2/3 to 1/3 ratio. Autologous bone was harvested from the crista zygomatico-alveolaris or retromolar for indications of lower jaw. The mesh was fixed to the residual bone with a titanium osteosynthesis screw (Bone Fixation Set®, Hager & Meisinger GmbH, Neuss, Germany) (Fig. 3b). A resorbable membrane (Bio-Gide®, Geistlich, Wolhusen, Switzerland) was placed on top. Flaps were adopted without tension by using deep mattress and single interrupted sutures (Mopylen 5–0; Resorba Medical GmbH, Nuremberg, Germany). In all cases, a slightly undermining preparation was sufficient to release the tension in the flap; an incision oft the periosteum could be avoided. Infection prophylaxis was ordered for seven days (Amoxicillin® 1000 mg 1–1–1), starting at time of the surgery. After a two-week primary healing period, sutures were removed. Postoperative care consisted of a clinical check-up every two months, instructions for normal oral hygiene and follow-up visits in the event of complaints.

### Removal of titanium meshes and implant placement

The healing period of the titanium meshes was six months (Fig. 3c). Implant placement (Camlog Screw Line Promote Plus®, Camlog, Wimsheim, Germany) and removal of the titanium meshes were performed in the same surgical procedure (Fig. 3d). The virtual implant planning was carried out with the help of a special planning software (coDiagnostiX®, Dental Wings Inc., Montreal, Canada). Special focus was set on the implant alignment allowing a later screw-retained, one-piece reconstruction. During surgery, the surgical guide template was used as a drill guide (Fig. 3e).

### Prosthetic treatment

After the second healing period (further 6 months), the surgical exposure of implants in rolled flap technique with installation of healing abutments was performed [36, 37].

**Fig. 4:**
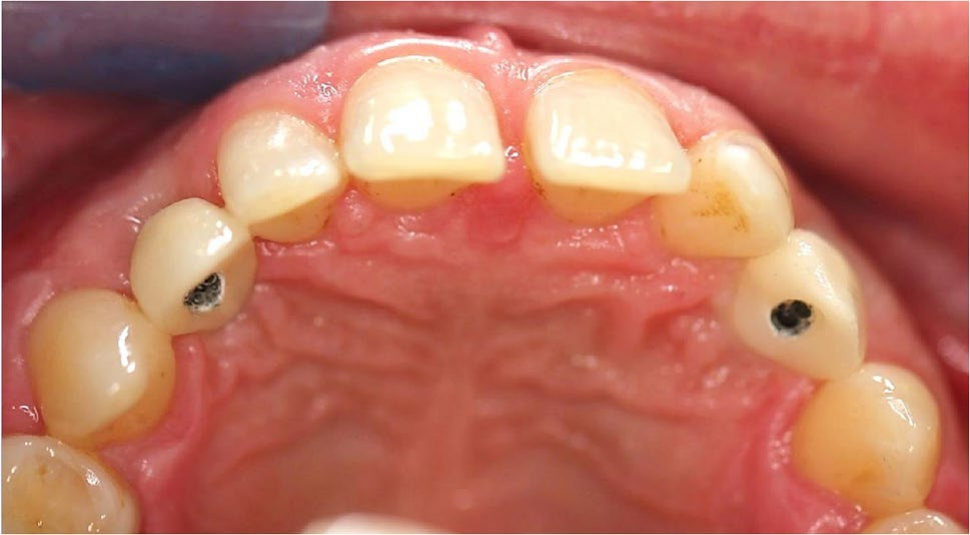
One-piece full-ceramic restorations (Imex®): in the anterior front with screw connection in the invisible palatal area, before semi-permanent underlayer and final acrylic material

Open impression method with polyether material was used (Impregum penta™, 3 M™, Espe™, Seefeld, Germany). One-piece full ceramic crowns (imex ®, imex dental group, Essen, Germany) with oral opening for the fixation screw was inserted with torque-control of 20 Ncm^−1^ (Fig. 3f/Fig. 4) The screw channels were filled with semi-permanent filling material as an underlayer (Cavit™-G, 3 M™, Espe™, Seefeld, Germany) and an acrylic filling to the upper definitive closure (Tetric EvoCeram, Ivoclar Vivadent AG, Schaan, Liechtenstein).

## Results

### Primary outcome variables

The results of the three-dimensional analysis and the linear measurements are listed in Table 1 for each case and illustrated in Figs. 6, 7 and 8.

**Table 1:** Analysis of the cross-sectional views of case series 1—3 for the linear measurement of concavity as well as for measuring the horizontal bone gain after 6 (first healing period) and 12 months (second healing period)and primary outcome variables (average ± standard deviation)

Patient and region	Buccal concavity baseline (mm)	Augmentation after 6 months (mm)	Augmentation after implantation, 12 months (mm)
***Patient 1***			
*013*	5.5	4.8	5.0
*023*	5.8	4.1	5.0
***Patient 2***			
*32*	3.3	3.5	4.8
*42*	3.3	3.1	5.1
***Patient 3***			
*34*	3.1	3.7	3.8
*32*	3.6	3.2	3.2
*41*	3.5	3.4	**3.4**
***Outcome variables***	**4.0 (*SD* ± 1.13)**	**3.7 (*SD* ± 0.59)**	**4.3 (*SD* ± 0.83)**

**Fig. 5:**
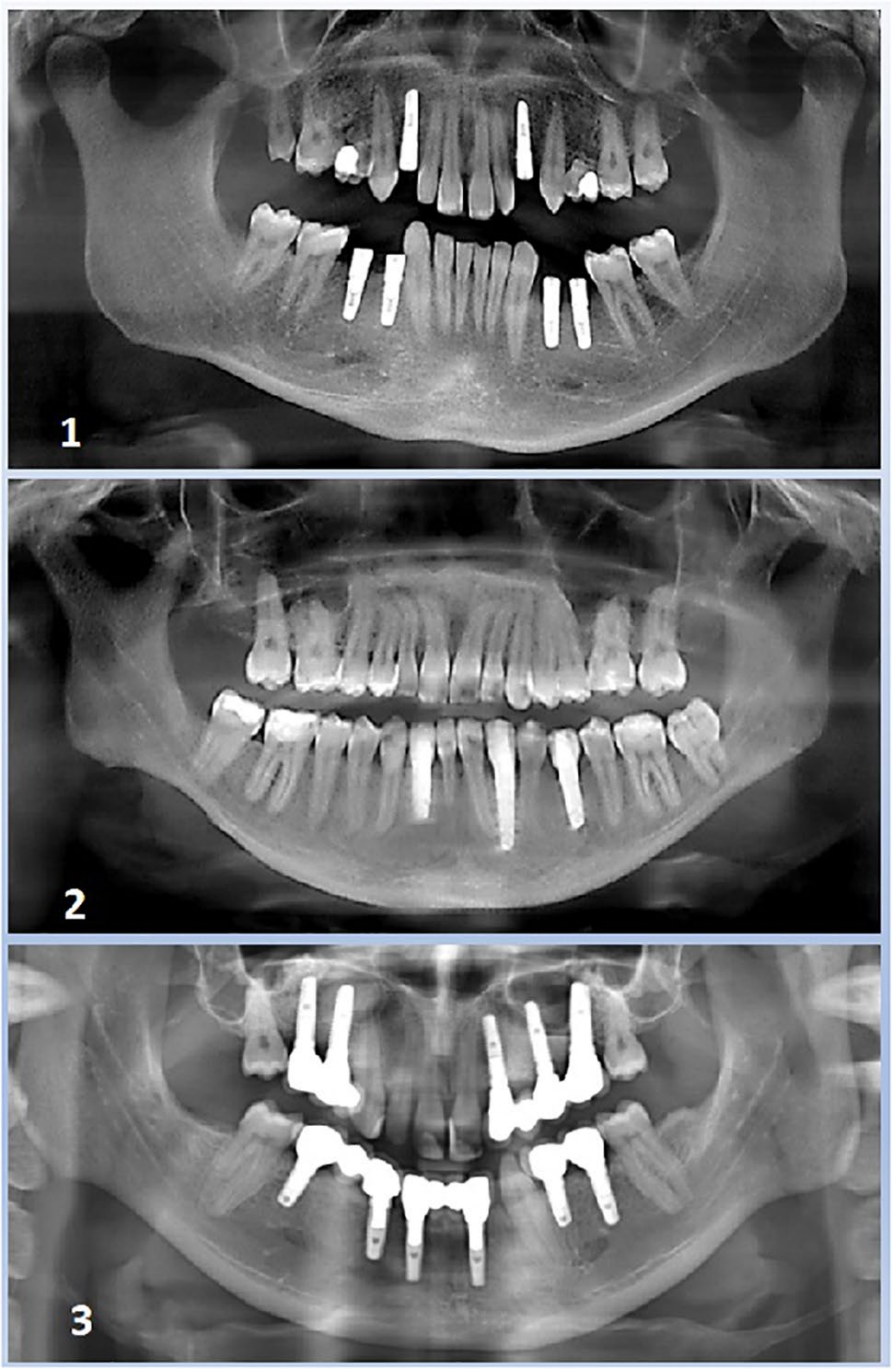
Panoramic views to the case series (patient 1–3): successful healing result for the seven implants patient 1 in the canine region (tooth number 13, 23 (FDI)), patient 2 with implants in the three single tooth gaps in the anterior lower jaw (34, 32, 42), patient 3 with two implants in the wide dental gap in anterior lower jaw (32, 42)

**Fig. 6:**
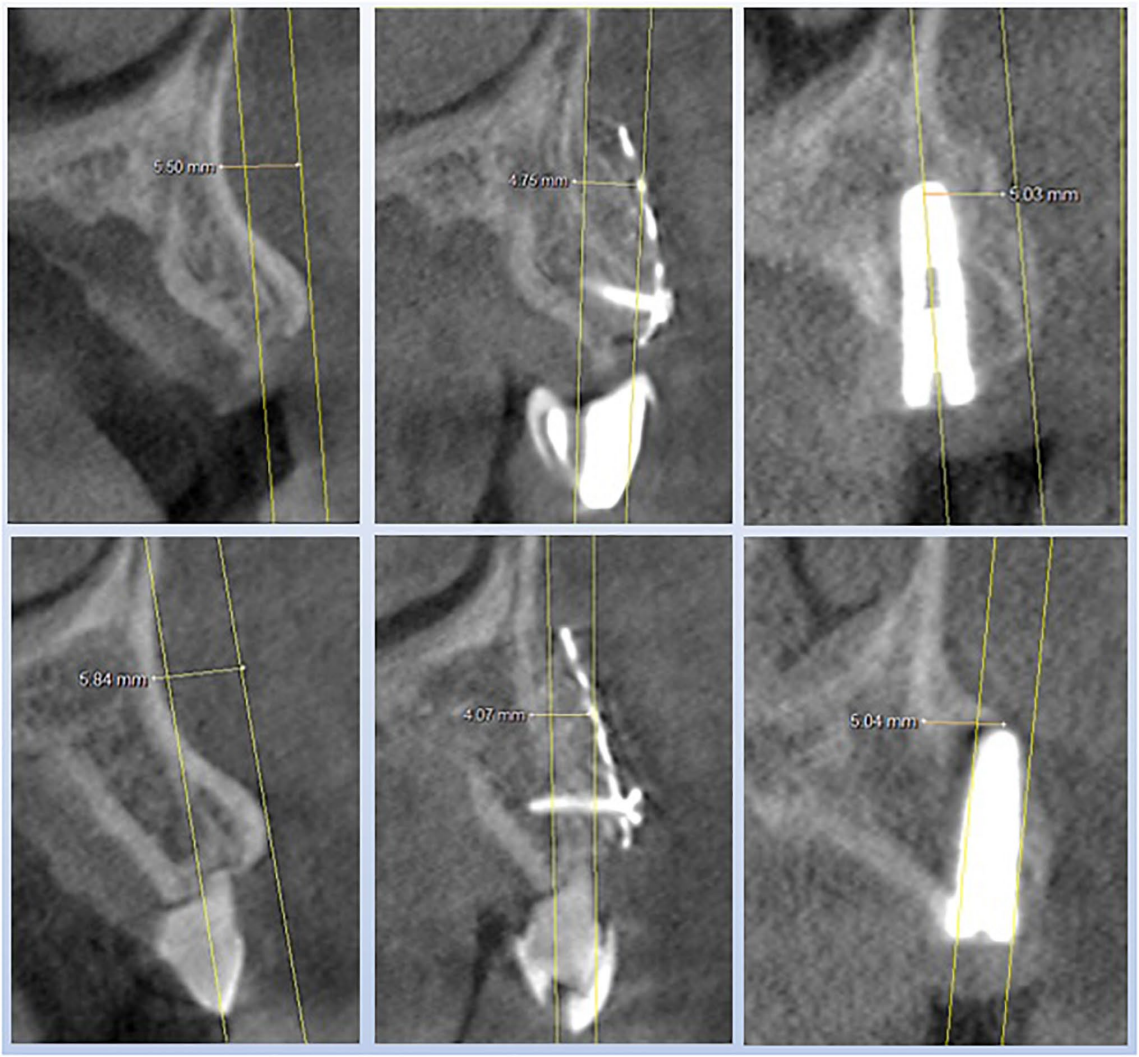
Analysis of the cross-sectional views of patient 1 with implants in region 13 (upper part) and 23 (lower part). On the left the images of the baseline measurement, in the middle the result of the bone augmentation after 6 months and on the right after another 6 months

**Fig. 7:**
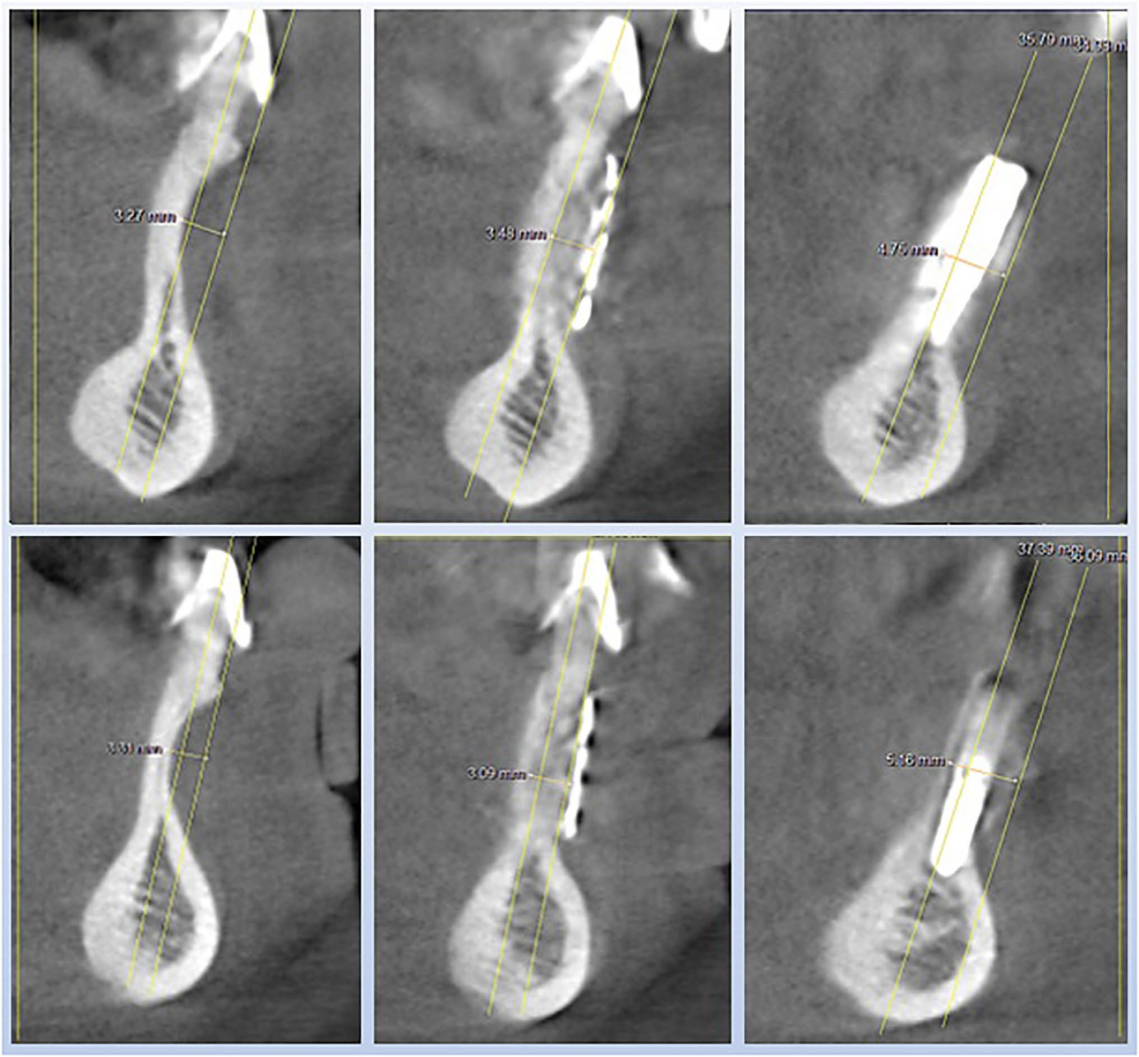
Analysis of the cross-sectional views of patient 2 with implants in region 32 (upper part) and 42 (lower part). On the left the images of the baseline measurement, in the middle the result of the bone augmentation after 6 months and on the right after another 6 months

**Fig. 8:**
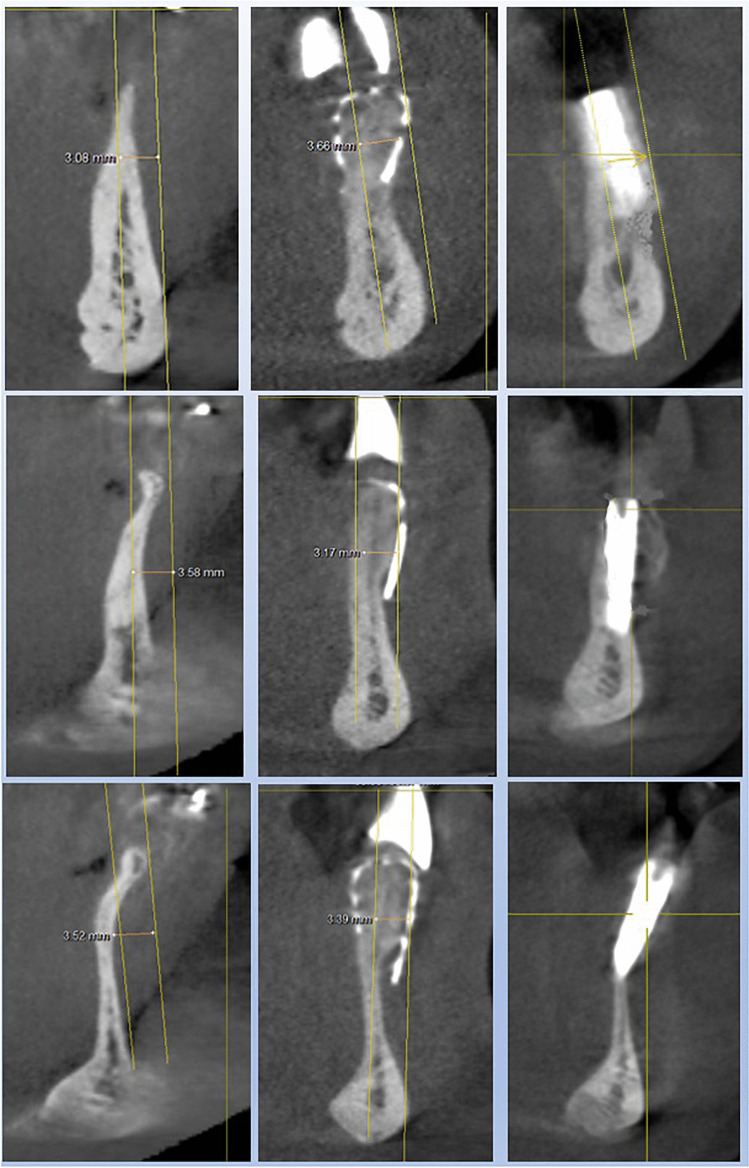
Analysis of the cross-sectional views of patient 3 with implants in region 34 (upper part) and 32 (middle) and 42 (lower part). On the left the images of the baseline measurement, in the middle the result of the bone augmentation after 6 months and on the right after another 6 months

For the seven cases recorded, the average value of the preoperative concavity depth was 4.0 mm (*SD* + / − 1.13). After augmentation with 3D-based titanium meshes (stage 1, after 6 months), an average of 3.7 mm of bone gain had to be measured (*SD* + / − 0.59), while after healing of the implants (stage 2, after 12 months), there was 4.3 mm (*SD* + / − 0.83) bone gain (Tab. 1) (Figs. 5, 6, 7 and 8).

Panoramic x-rays document the course the successful healing result of the case series (Fig. 5). In all cases, there is a regular bone course, without vertical bone loss mesially and distally.

### Secondary outcome variables

During the healing period of augmentation, no exposure of titanium meshes could be observed. Only in two cases the mucosa appeared somewhat thinned, so that the mesh shimmered through partially (Fig. 3c). No signs of inflammation could be detected during healing. After the healing period, the bone augmentation could be used in all cases for implantation. In six of the seven cases, the screw hole was in a palatal position so that a screw-retained reconstruction could be fixed on the implants. Due to the implant axis, one case had to be treated with a cemented two-part full-ceramic system.

## Discussion

**Fig. 9:**
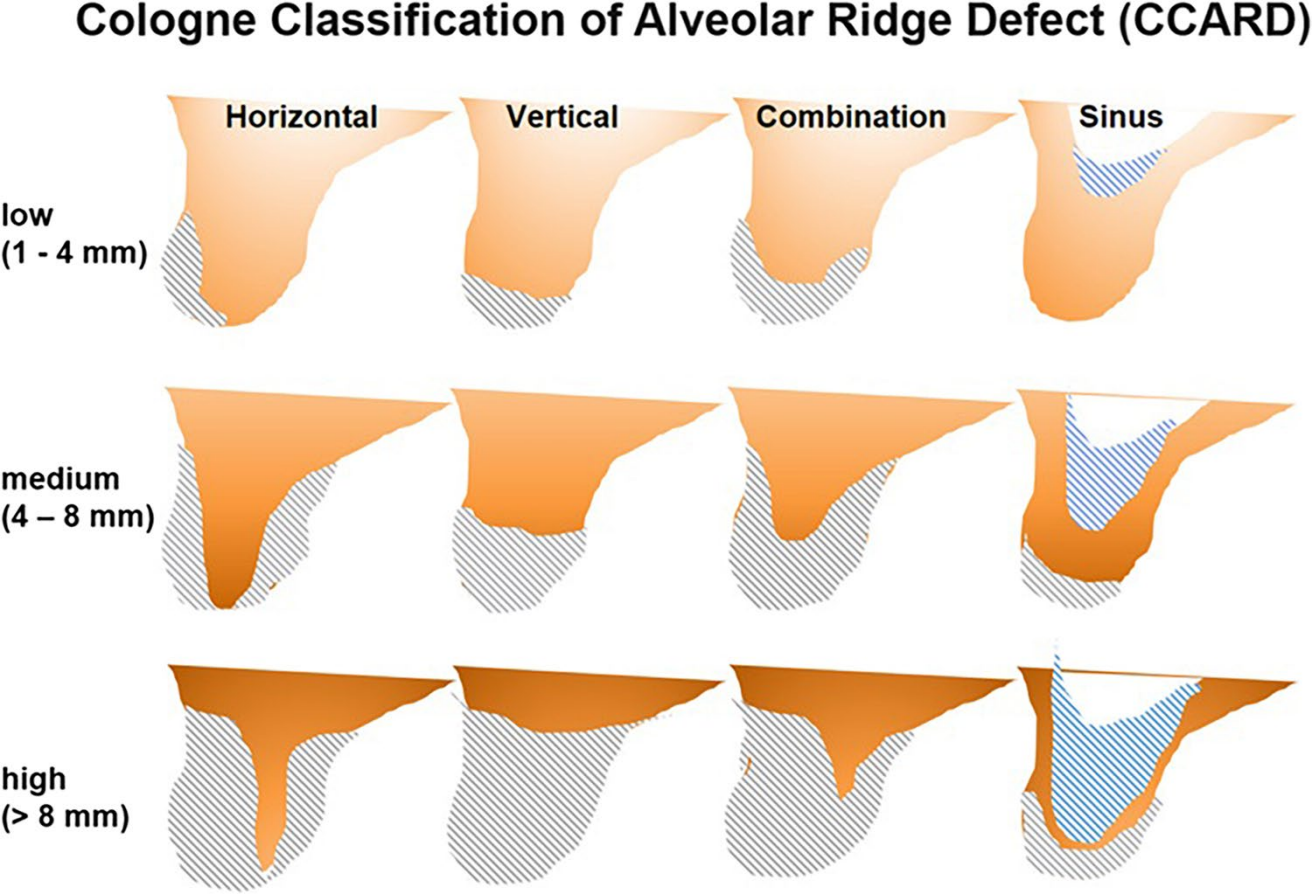
Cologne Classification of Alveolar Ridge Defects (CCARD) differentiates between horizontal, vertical and combined defects of different dimensions (less than 4 mm/up to 8 mm/more than 8 mm)

To select different bone augmentation procedures, the alveolar bone defect should be examined in detail. Based on the Cologne classification of the alveolar ridge defect (CCARD), the alveolar ridge defects can be divided into horizontal, vertical and combined defects of different dimensions (less than 4 mm/up to 8 mm/more than 8 mm) (Fig. 9). The CCARD also gives recommendations regarding suitable augmentation methods, depending on the type and extent of the defect (Table 2). According to systematic reviews [11–13], it seems feasible for GBR with titanium meshes to achieve an average bone gain of 3 to 4 mm in width and height. In accordance to this, the CCARD describes the indication for GBR with titanium mesh for horizontal defects up to a maximum of 8 mm and for vertical or combined defects up to a maximum of 4 mm distance. The case series presented corresponds to the indication for horizontal defects up to 4 and 4 to 8 mm.

**Table 2:** CCARD also gives recommendations regarding suitable augmentation methods, depending on the type and extent of the defect

Cologne Classification of Alveolar Ridge Defect (CCARD) and augmentation methods	Horizontal < 4 4–8 > 8			Vertical < 4 4–8 > 8			Combined < 4 4–8 > 8		
***Internal the ridge***									
Guided bone regeneration (GBR)	X			x			x		
***GBR with titanium mesh***	**x**	**x**		**x**	**(x)**		**x**		
Distraction osteogenesis				x	x	x	x	x	
Onlay graft				x	x	x	x	x	x
Bone splitting/ridge expansion	x	x					x		
***External the ridge***									
Guided bone regeneration (GBR)	x								
***GBR with titanium mesh***	**x**	**(x)**		**x**					
Distraction osteogenesis				x	x	x			
Onlay graft	x	x	x	x	x	x	x	x	x
Bone splitting/ridge expansion	x	x					x		

The main nonabsorbable membranes are expanded polytetrafluorethylen (ePTFE), titanium-reinforced PTFE, and titanium mesh [7]. Among these, titanium mesh is the only one entirely made of metal. It exploits the advantages of titanium in mechanical and biological properties to the full, performing excellent in space maintenance and bone reconstruction [13]. Nonetheless, study results show that there is no significant difference between titanium meshes and titanium-reinforced PTFE in terms of vertical bone augmentation and complication rates [38, 39].

According to the currently published literature, GBR with titanium mesh has favorable predictability of osteogenesis in both delayed and simultaneous implantation. With the delayed implantation strategy after bone augmentation, most researchers have achieved an average bone augmentation of 5–6 mm in bone width and height [14]. With the strategy of simultaneous implantation with bone augmentation, the realization of 3–4 mm average bone gain in width and height appears somewhat reduced in comparison [18]. In the present case series, we decided for a delayed implantation strategy and the average bone gain was around 4–5 mm for the horizontal deficit.

Compared to other methods, bone loss due to infection is rare in the application of titanium meshes [39]. Peri-implant bone resorption has been reported, which usually occurs during implant healing [18, 40]. In the present case series, there was no evidence of bone loss after implant placement or after 6-month healing period of implants.

Some studies have tried to investigate the relationship between the pore size and the extent of soft tissue growth. Whether the pore size of the titanium meshes has a relevant influence on the extent of regenerated bone cannot be conclusevly assessed [41]. In our observational study, the titanium meshes used had a large pore size of 2 mm. Some authors observed, that after alveolar ridge reconstruction conducted by titanium meshes with wide pores, a thin layer of 1–2 mm thick, soft tissue can often be found upon the regenerated bone surface, called “pseudo-periosteum” [38]. The formation of this soft tissue layer may be related to the insufficient cell exclusion ability of meshes due to its pores. The role of pseudo-periosteum may be related to bone graft protection, graft infection prevention, and absorption [42]. Accordingly, we could not record any infections or other complications in our case series.

Many clinical studies showed that a customized titanium mesh plays an active role in shortening operation time, reducing the occurrence rate of bone augmentation complications, and improving the success rate of surgery [38, 43]. The main reason for the advantages mentioned is the considerably more favorable fit compared to conventional meshes. The titanium meshes used in this observational study were all characterized by a perfect fit.

A typical horizontal defect situation occurs with bony concavities of the alveolar bone and is of great relevance for implant prosthetics, especially in the anterior region. If the concavity of buccal bone is not treated, optimal alignment of implant is not possible and the preferred screw-retained full-ceramic restoration is not possible. The opening for the fixation screw of the restoration should preferably be placed in the invisible oral area [44, 45], which is only possible if the implant can be placed in a suitable position and angle. With the alternative of a two-part, cemented restoration, the margin of fixed partial denture often is visible [26]. Screw-retained reconstructions also show fewer biological problems such as implant failure or marginal bone loss than cement-retained reconstructions [24]. Linkevicius et al. [46] found a prevalence of peri-implant disease in 0.8% of the screw-retained restorations, while 75% of the implants provided with cemented restorations were diagnosed for peri-implant disease, 64% of those being positive for cement excess. With the optimized method, six out of seven cases could be treated with screw-retained restoration.

The orofacial position of the implant shoulder is strongly associated with mucosal recession, especially following immediate implant placement [47, 48]. In a retrospective study of the esthetic outcomes, Evans and Chen [49] found that implants with a buccal shoulder position showed three times more recessions than implants with an oral shoulder position. Cosyn et al. reported that the buccal shoulder position increased the likelihood of mid-buccal recession (odds ratio = 17.2) [48]. The more buccal the position of the implant, the more the midbuccal margin recedes apically [50]. Likewise, a more proclined implant position and an increased depth of the implant platform significantly increase the risk of buccal recession defects. With the method shown in our case report, the implant can always be placed in an ideal three-dimensional position, which reduces the risk of mucosal recession.

## Conclusions

With the described optimized method esthetically demanding screw-retained, full ceramic restoration which is biologically superior can also be used in situations with unfavorable concavities of buccal bone. Especially for this indication, a special form of the horizontal deficit, the customized bone regeneration with titanium meshes is highly reliable in terms of healing and extent of augmentation. However, long-term results and a study/control group are required to evaluate the effectiveness of the presented protocol.
